# Supplementary material for: Direct RNA Nanopore Sequencing of SARS-CoV-2 Extracted from Critical Material from Swabs
Source: Life (Basel). 2022 Jan 4;12(1):69. doi: 10.3390/life12010069 (PMC8778588; doi:10.3390/life12010069)
Supplement: Supplementary file 1 [file life-12-00069-s001.zip › life-1454229-supplementary.pdf]

life

Supplementary Material of Direct RNA Nanopore Sequencing of SARS-CoV-2 Extracted from Critical Material from Swabs

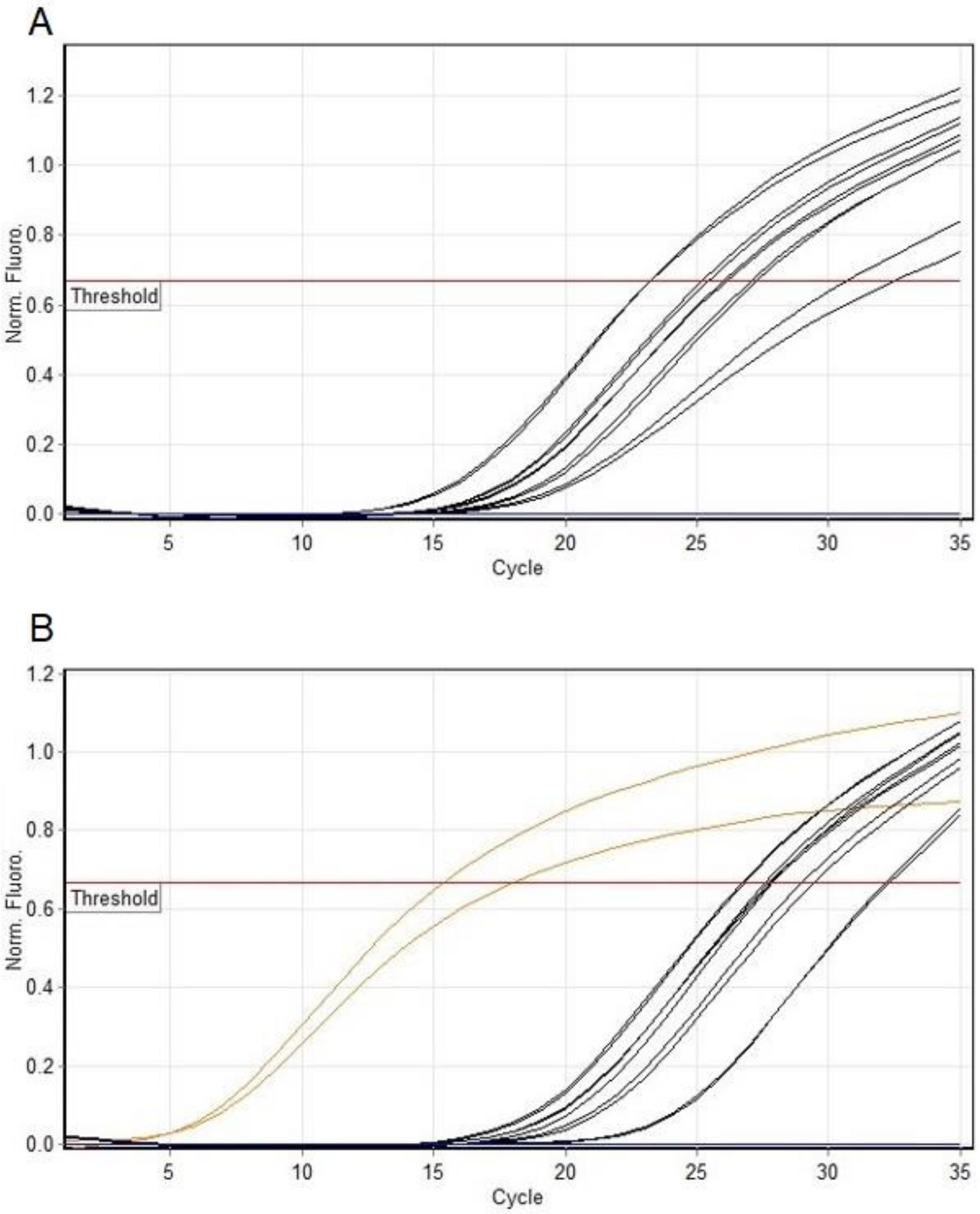

Figure S1. Amplification curves in the qPCR assay with primer set N2 from 2019-Novel Coronavirus (2019-nCoV) Real-time rRT-PCR Panel, from IG1 to IG5 (A) and from IG6 to IG10 with negative and positive control samples (B), as reported in the Materials and Methods. The IG and N reference samples tested positive in the assay compared to the brain samples. Relative Ct values are reported in Table S2.

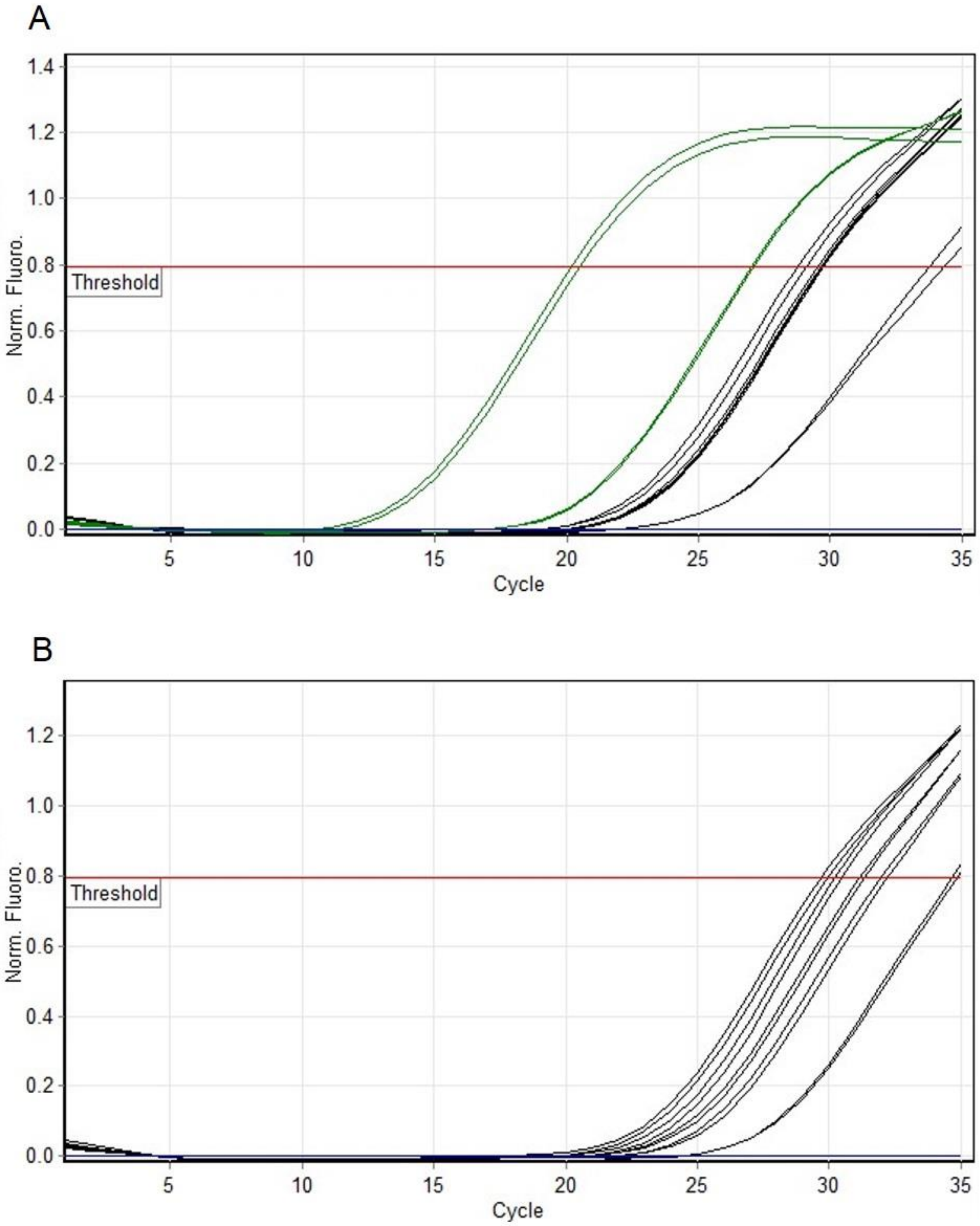

Figure S2. Amplification curves in the qPCR assay with primer set specific for Human housekeeping GAPDH, from IG1 to IG5 (A) and from IG6 to IG10 with negative and positive control samples (B), as reported in the Materials and Methods. The IG and brain samples tested positive in the assay compared to the synthetic SARS-CoV-2 N gene. Relative Ct values are reported in Table S3.

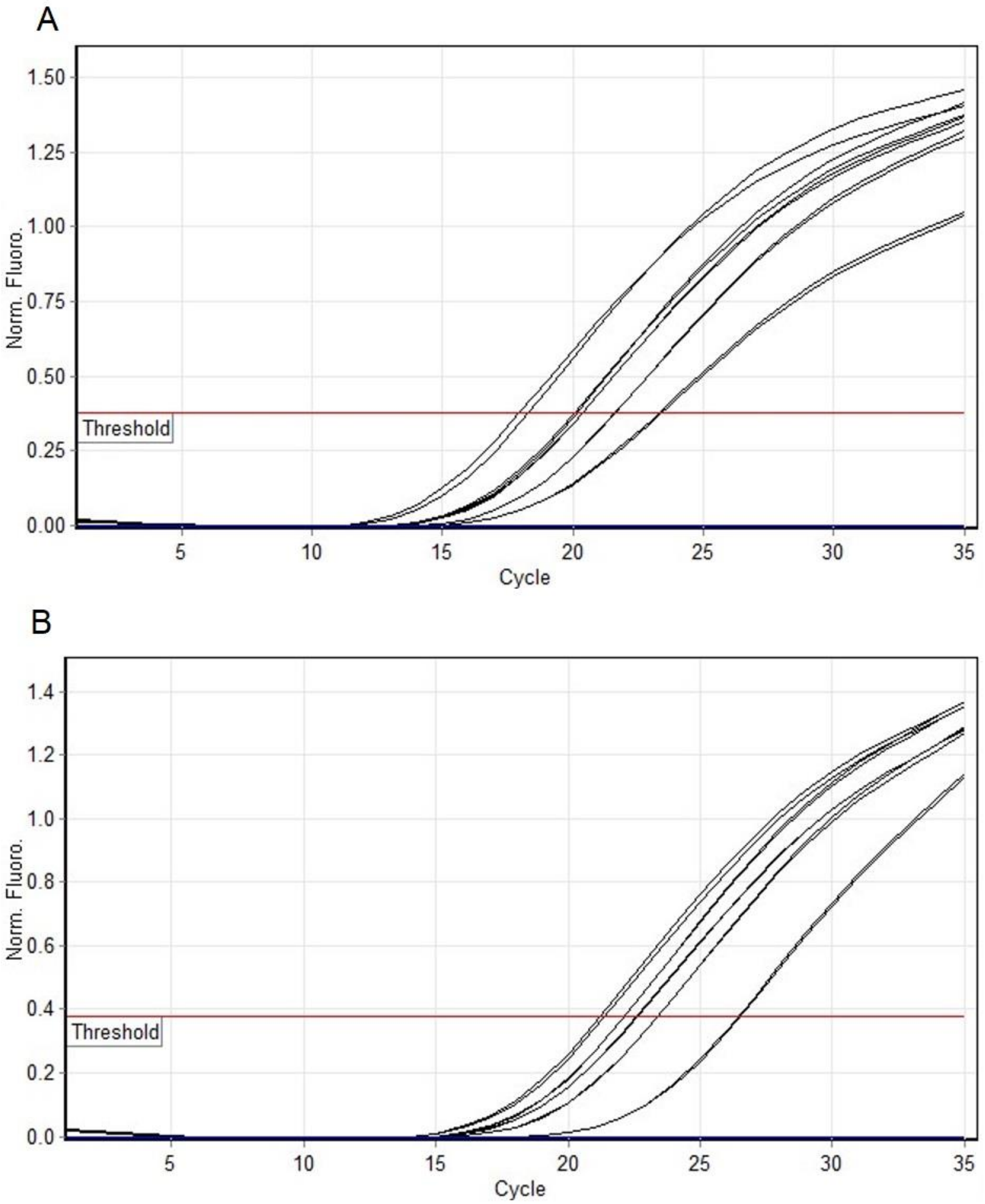

Figure S3. Amplification curve in the qPCR assay with the primer set specific for the mutated region, from IG1 to IG5 (A) and from IG6 to IG10 with negative and positive control samples (B), as reported in the Materials and Methods. All the IG samples exhibited mutation (frequency of 100%) compared to the reference and brain biopsy samples. Relative Ct values are reported in Table S5.

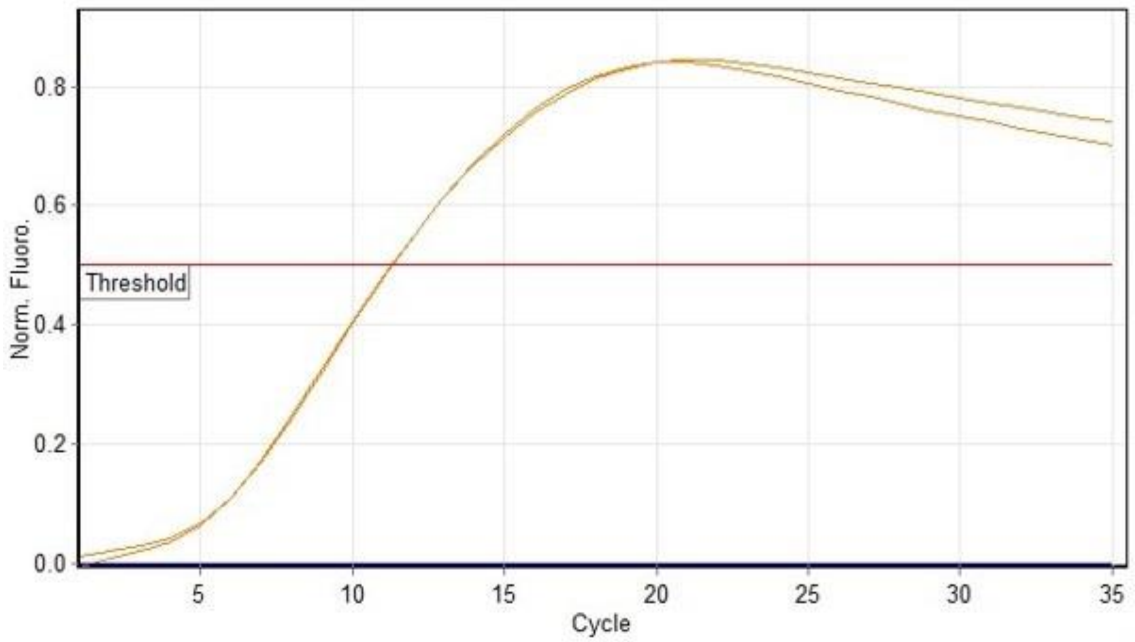

Figure S4. Amplification curves in the qPCR assay for samples using the WT primer set. The plasmid vector carrying a synthetic reference SARS-CoV-2 N gene was the unique sample positive compared to other samples, as reported in the Materials and Methods. Relative Ct values are reported in Table S6.

Table S1. The primer sequences of the sets used for the enrichment of the mutated region in the Sanger sequencing workflow, and of the primers specific for the analyzed targets in the real-time PCR experiment, as reported in the Materials and Methods.

Primer Set Name	Primer Sequence (5′ → 3′)	Size (bp)
Sanger_nCoV_Gene N_OUT	F: CAAGGCGTTCCAATTAACA<br>R: CAGCAGGAAGAAGAGTCA	985
Sanger_nCoV_Gene N_INN	F: TACGCAGAAGGGAGCAGAG<br>R: AGCAGCAAAGCAAGAGCA	155
2019-nCoV_N2	F: TTACAAACATTGGCCGCAAA<br>R: GCGCGACATTCCGAAGAA	67
2019-nCoV_28881_28882_28883-N	F (wt): AACTCCAGGCAGCAGTAGGG<br>F (mut): AACTCCAGGCAGCAGTAAAC<br>R: TTGGCCTTGTTGTTGTTGGC	142
GAPDH (NM_002046.7)	F: GATTTGGTCGTATTGGGCGC<br>R: CATGTAAACCATGTAGTTGAGGTCA	110

Table S2. CT values in the qPCR assay for samples derived from: swabs collected from ten SARS-CoV-2-positive patients (from IG1 to IG10); brain biopsy (n7 and n9); synthetic reference N gene and No template controls (ntc), with primer set N2 from 2019-Novel Coronavirus (2019-nCoV) Real-time rRT-PCR Panel, as reported in the Materials and Methods.

CT Values–Threshold 0.66697			
Color	Name	Type	Ct
	ig1	Unknown	32.49
	ig1	Unknown	30.65
	ig2	Unknown	23.23
	ig2	Unknown	23.22
	ig3	Unknown	25.38
	ig3	Unknown	25.17
	ig4	Unknown	25.97
	ig4	Unknown	26.07
	ig5	Unknown	27.02
	ig5	Unknown	27.24
	n7	Unknown	
	n7	Unknown	
	n9	Unknown	
	n9	Unknown	
orange	N reference	Unknown	17.95
orange	N reference	Unknown	15.38
	ig6	Unknown	27.89
	ig6	Unknown	27.62
	ig7	Unknown	26.79
	ig7	Unknown	26.73
	ig8	Unknown	32.32
	ig8	Unknown	32.16
	ig9	Unknown	27.84
	ig9	Unknown	27.76
	ig10	Unknown	29.01
	ig10	Unknown	29.45
blue	ntc	Unknown	
blue	ntc	Unknown	

Table S3. CT values of the qPCR assay for samples derived from: swabs collected from ten SARS-CoV-2-positive patients (from IG1 to IG10); brain biopsy (n7 and n9); synthetic reference N gene and No template controls (ntc), with primer set specific for Human housekeeping *GAPDH*, as reported in the Materials and Methods.

CT Values–Threshold 0.79373			
Color	Name	Type	Ct
	ig1	Unknown	34.33
	ig1	Unknown	33.77
	ig2	Unknown	29.66
	ig2	Unknown	29.55
	ig3	Unknown	29.73
	ig3	Unknown	29.77
	ig4	Unknown	28.79
	ig4	Unknown	29.11
	ig5	Unknown	29.68
	ig5	Unknown	29.71
green	n7	Unknown	20.15
green	n7	Unknown	20.49
green	n9	Unknown	27.08
green	n9	Unknown	27
	N reference	Unknown	
	N reference	Unknown	
	ig6	Unknown	30.23
	ig6	Unknown	30.48
	ig7	Unknown	31.16
	ig7	Unknown	31.33
	ig8	Unknown	34.81
	ig8	Unknown	34.63
	ig9	Unknown	29.71
	ig9	Unknown	29.96
	ig10	Unknown	31.95
	ig10	Unknown	32.19
blue	ntc	Unknown	
blue	ntc	Unknown	

Table S4. Result of the reads alignment process given by the bioinformatics pipeline.

	Number of Reads	Percentage of Good-Quality Reads
Reads from MinION sequencing technlogy	397465	
Good-quality reads (quality > 8, length > 500nt)	20940	100%
Fungi (Saccharomyces cerevisiae)	20351	97.19%
Homo Sapiens (Chromosome MT)	93	0.44%
SARS-CoV-2	54	0.26%
Bacteria	0	0%
ND	442	2.11%

Table S5. Ct values in the qPCR assay for samples derived from: swabs collected from ten SARS-CoV-2-positive patients (from IG1 to IG10); brain biopsy (n7 and n9); synthetic reference N gene and No template controls (ntc), with the primer set specific for the mutated region.

CT Values–Threshold 0.37927			
Color	Name	Type	Ct
	ig1	Unknown	23.31
	ig1	Unknown	23.43
	ig2	Unknown	18.34
	ig2	Unknown	17.97
	ig3	Unknown	20.16
	ig3	Unknown	20.08
	ig4	Unknown	20.38
	ig4	Unknown	20.37
	ig5	Unknown	21.65
	ig5	Unknown	21.65
	n7	Unknown	
	n7	Unknown	
	n9	Unknown	
	n9	Unknown	
	N reference	Unknown	
	N reference	Unknown	
	ig6	Unknown	22.18
	ig6	Unknown	22.18
	Ig7	Unknown	21.44
	Ig7	Unknown	21.26
	ig8	Unknown	26.46
	ig8	Unknown	26.54
	ig9	Unknown	22.67
	ig9	Unknown	22.63

	ig10	Unknown	23.42
	ig10	Unknown	23.43
blue	ntc	Unknown	
blue	ntc	Unknown	

Table S6. Ct values in the qPCR assay for samples derived from: swabs collected from ten SARS-CoV-2-positive patients (from ig1 to ig10); brain biopsy (n7 and n9); synthetic reference N gene and No template controls (ntc), using the WT primer set.

CT Values–Threshold 0.50197			
Color	Name	Type	Ct
	ig1	Unknown	
	ig1	Unknown	
	ig2	Unknown	
	ig2	Unknown	
	ig3	Unknown	
	ig3	Unknown	
	ig4	Unknown	
	ig4	Unknown	
	ig5	Unknown	
	ig5	Unknown	
	n7	Unknown	
	n7	Unknown	
	n9	Unknown	
	n9	Unknown	
orange	N reference	Unknown	11.28
orange	N reference	Unknown	11.30
	ig6	Unknown	
	ig6	Unknown	
	ig7	Unknown	
	ig7	Unknown	
	ig8	Unknown	
	ig8	Unknown	
	ig9	Unknown	
	ig9	Unknown	
	ig10	Unknown	
	ig10	Unknown	
blue	ntc	Unknown	
blue	ntc	Unknown	
